# Feeling the heat: Temperature and fertilizer's role in cooking up a high yielding raspberry crop (***Rubus idaeus***) grown in a controlled, indoor, hydroponic environment

**DOI:** 10.1177/18785093251326169

**Published:** 2025-05-22

**Authors:** Anna Bazangeya, Serena Sbrizzi, Huzaifa Almohimed, Yoana Angelova, Habiba Bougherara, Lesley G Campbell

**Affiliations:** 1 Department of Chemistry and Biology, Toronto Metropolitan University (recently renamed), Toronto, Canada; 2 Department of Mechanical and Industrial Engineering, Toronto Metropolitan University (recently renamed), Toronto, Canada

**Keywords:** controlled environment agriculture, air temperature, yield, sugar content, endomycorrhizae, fertilizer, berry, fruit quality

## Abstract

**Background:**

Temperature and fertilizer crucially influence fruit quality. While well-studied for outdoor-grown red raspberries, optimal conditions for controlled indoor agriculture are less understood.

**Objectives:**

This study aimed to identify the best temperature and fertilizer regimen to maximize fruit production, sweetness, and harvest index in an indoor, hydroponic vertical farm.

**Methods:**

We tested three temperatures (21, 23, 25°C) and three fertilizer mixes (A: weak fertilizer applied at a constant rate, B: developmentally adjusted fertilizer (DAF) and C: DAF plus commercial endomycorrhizal fungi) on ‘Joan J’ raspberries in a controlled indoor hydroponic vertical farm in Toronto, Canada. We measured fruit number, weight, and sugar content.

**Results:**

Raspberries grown at 23°C produced significantly more (∼30%) total fruit biomass than those at 21 and 25°C (F = 17.19, P<0.001). Fruit weight was higher earlier in the season, decreasing by 29% in the following three months. Temperature and time interacted such that the largest fruit was produced at 21°C in the first month (F = 3.70, P < 0.001). Fertilizer B yielded significantly greater (26–35%) more fruit and harvest index than Fertilizers A or C (F=5.16, P<0.001), though no significant differences were found in the interaction between fertilizer and time. Additionally, raspberries grown at 23°C had significantly higher sugar content (9.89°Bx, P < 0.05) compared to other temperatures, but fertilizer did not influence sweetness.

**Conclusions:**

While 21°C yielded the most fruit early in the season, 23°C produced the highest overall yield and sweetest fruit, lower than typical outdoor conditions for temperate climate raspberries. Developmentally adjusted fertilizers increase raspberry yield.

## Introduction

Red raspberry is an economically and culturally important fruit crop in many countries, including Canada, known for its flavor and health benefits.^1,2^ However, due to the seasonal characteristics of raspberry cultivation, Canada's short growing season and variable weather conditions, a significant amount of Canada's raspberry consumer demand is met through imports.^3–5^ Most of the Canadian commercial red raspberry production occurs in British Columbia (71%), Quebec (18%), and Ontario (7.5%), using a variety of methods such as outdoor farming, greenhouses, and high-tunnel soil-less systems.^3,6^ Further, only a small portion (approximately 7%) of Canada's land is suitable for raspberry crop production^7^ since most raspberry cultivars typically grow in USDA zones 3–9.^8^ With climate change rapidly impacting agricultural production,^9^ there is a need for Canada to develop sustainable solutions for year-round raspberry cultivation.

The optimization of red raspberry growth and fruiting is well studied. Much of the recent research has focused on improving cultivation methods and extending the length of the growing season.^10–12^ The development and implementation of soilless high tunnels, plastic and heated greenhouses, and pruning techniques have made a significant impact on raspberry production.^13–15^ Year-round cultivation of red raspberries is possible in warmer Mediterranean climates using unheated plastic greenhouses.^11^ Nevertheless, year-round growth in temperate climates has not yet been achieved.

Controlled environment agriculture (CEA) allows for automated and precise control of water, nutrients, air temperature, humidity, photoperiod, and light intensity based on specific plant requirements, while mitigating the impacts of external weather conditions.^16^ In particular, hydroponics horticulture of raspberries and other plants allows farmers to reduce water and pesticide usage, eliminate soil-borne infections, and increase yield per unit area.^17,18^ For instance, in high tunnels, soilless substrates such as coco coir have been found to increase yield compared to soil in red raspberries.^12,19^ Yet, the vast majority of this research has been conducted in environments that are influenced by outdoor weather conditions (light intensity, photoperiod, temperature, etc). To achieve year-round, high-volume raspberry production, we must measure the influence of precise environmental conditions, particularly temperature and nutrient application, on raspberry fruit biomass, fruit number, and fruit quality.

Optimal temperature requirements for maximizing fruit yield vary at each stage of development. Temperature has a significant and varying effect on yield and fruit quality, depending on whether the raspberry is annual- or biennial-fruiting, as well as the cultivar.^20^ Temperature also plays an important role in growth cessation and floral initiation in raspberries.^20,21^ The temperature that raspberries are exposed to prior to dormancy affects yield in the following season in biennial fruiting raspberries. Low temperatures of 9°C at the end of the growing season produce berries with a higher number of drupelets, and thus weight, in the early part of the following harvest season.^21^ This may be an effect of increased female sex expression in lower temperatures.^21^ While biennial-fruiting cultivars require low temperatures below 15°C for flower initiation, annual-fruiting plants have been shown to have no trouble fruiting at higher temperatures of 27°C and even 30°C.^10,20^ Regarding the number of fruit, many cultivars of annual-fruiting raspberries, such as ‘Erika’, ‘Polka’ and ‘Autumn Bliss’, seem to favor high temperatures during the first 5 weeks of growth post-dormancy, reflected in a higher percentage of flowering and fruiting nodes and earlier maturation. Nonetheless, some annual cultivars, specifically ‘Autumn Treasure’ have been shown to prefer lower temperatures of 20°C, with higher temperatures limiting yield. Furthermore, higher temperatures, while increasing the number of fruits produced, are correlated with reduced fruit weight in annual-fruiting raspberries.^20^ A similar pattern is seen in biennial-fruiting cultivar ‘Glen Ample’ where although high temperatures advance maturation and harvest, berry weight decreases.^21–23^ Growing ‘Glen Ample’ at lower temperatures of 12–18°C during the early fruiting stages results in higher fruit weight and Vitamin C content.^22^

Fertilization is crucial for optimal yield and has been extensively studied in red raspberry research.^21,23^ Most fertilizer recommendations are specifically for soil-grown raspberries, taking into consideration soil characteristics, plant age, weather conditions, irrigation, and cultivar.^8,24^ However, with the advent of hydroponic horticulture of raspberries in coco coir in high tunnels, this is changing.^12,19^ Nitrogen is particularly important for primocane growth early in the growing season, while floricane growth relies on stored nitrogen drawn from overwintering primocanes, crown, and root.^8^ Increasing nitrogen application (135 kg per ha divided and applied in 3 parts) increases the number and weight of fruit, resistance to mechanical damage, firmness, and fruit quality, but decreases dry matter content for biennial fruiting cultivars ‘Glen Ample’ and ‘Laszka’.^23^ In soilless substrate systems, biennial raspberry yields have also benefited from 3 g/L osmocote fertilizer with additional fertilization during the first six weeks of propagation.^19^ Interestingly, it is possible that when plants are provided adequate nutrients up to the floral initiation stage, the reserves are enough for acceptable floral differentiation and decreasing fertilization overtime does not affect yield and berry size.^21^ In annual-fruiting ‘Meeker’ one study found no difference in yield between fertilizer treatments in the first year.^25^ However, yield and fruit weight increased in the second year with split fertilizer application of 40 kg/ha of nitrogen at budbreak and 40 kg/ha of N applied eight weeks later in outdoor soil systems. Using deep-water culture hydroponic, one study found that annual fruiting cultivar ‘Redwing’ exhibited a higher number of nodes, flowers and inflorescences per cane when submerged in concentrations of 5, 10, and 20-meq nitrogen/L from the time of propagation..^26^ Treftz & Omaye^18^ obtained larger berries in a hydroponic system than soil grown using General Hydroponic Flora Series (500 ppm). In the field, raspberries can also form symbiotic relationships with Arbuscular mycorrhizal fungi (AMF) to improve nutrient uptake, growth, disease resistance and stress tolerance.^27–29^ Raspberry plants inoculated with AMF increased fruit yield by 43%.^27^ This may be crop and AMF species dependent; since various AMF isolates have demonstrated neutral or negative effects in other crops.^28^

Given this background, the aim of this research is to study the effect of temperature and fertilizer schedule on the raspberry fruit quality of annual fruiting ‘Joan J’ cultivar grown in a controlled, indoor hydroponic system. Based on the literature review, we hypothesized that within a controlled, indoor hydroponic setting, lower temperatures of 21°C and the addition of AMF will confer enhanced raspberry fruit productivity. This enhancement is expected in contrast to the elevated temperatures of 23°C and 25°C and a constant rate of fertilizer, reflected through increased mean individual and total fruit biomass, as well as the number of fruits yielded per individual plant. By leveraging existing research on temperature's influence on raspberry phenology, this article contributes to the development of guidelines for successful raspberry production within CEA systems. This knowledge is essential for advancing sustainable agriculture practices and ensuring year-round fruit supply. This paper addresses two main research questions: What is the effect of temperature on raspberry production using the Joan J cultivar? and How do fertilizers recipes influence raspberry fruit production and quality? The paper also documents the production of raspberry fruit in a constant environment, without a seasonal change in weather. In answering these questions, we address a research gap in the limited understanding of optimal temperature and fertilizer regimens for maximizing raspberry yield and quality in fully controlled indoor hydroponic systems. While raspberry cultivation has been extensively studied in outdoor and high-tunnel environments, the precise conditions required for year-round production in controlled environment agriculture (CEA) remain largely unexplored.

Our results demonstrated red raspberry plants cv. ‘Joan J’ achieved the highest fruit yield and harvest index at relatively low temperatures, temperatures which could only be achieved using Controlled Environment Agriculture (CEA) year-round. CEA, through its ability to carefully adjust environmental conditions, may be a tool for successful year-round raspberry production through the control of temperature and nutrient requirements to maximize the quantity and quality of red raspberry ‘Joan J’ fruit produced in a vertical hydroponic farm.

## Materials and methods

### Study system

The annual-fruiting red raspberry (*Rubus idaeus* L.) cultivar ‘Joan J’ was propagated in our system from bare root plants (Lareault Nursery, Lavaltrie, QC, Canada). The cultivar is a product of crossing 'Joan Squire' and 'Terri-Louise' in 1993, and is characterized by medium red, broad conical berries of medium firmness, supported by plants with medium green foliage.^30^ We selected this cultivar for its early fruiting period, relatively large fruit size and flavor, high leaf chlorophyll content, productivity, and growth habit. 'Joan J' is an early fruiting cultivar, suggesting it would be efficient in a vertical farm where it is important to minimize the time to fruiting.^31,32^

### Horticultural methods

The experiment was conducted between 2022 and 2023 at the Campbell Laboratory (43.66° N, −79.38° W) at Toronto Metropolitan University, Toronto/Tkaronto, Ontario, Canada. On June 8–10, 2022, rootstock plants were individually planted in 2-gallon pots (GrowerBasics, Mississauga, Canada) containing a 3:7 mixture of perlite and vermiculite (Holiday, Mississauga, Canada). The plants were initially grown at 50% humidity with gradually increasing light intensity (details in Appendix Table A1). After 17 days, plants received full light-exposure of 195 μmol/m2/s (90 Cool White + 105 Dark Red).

**Figure 1.**
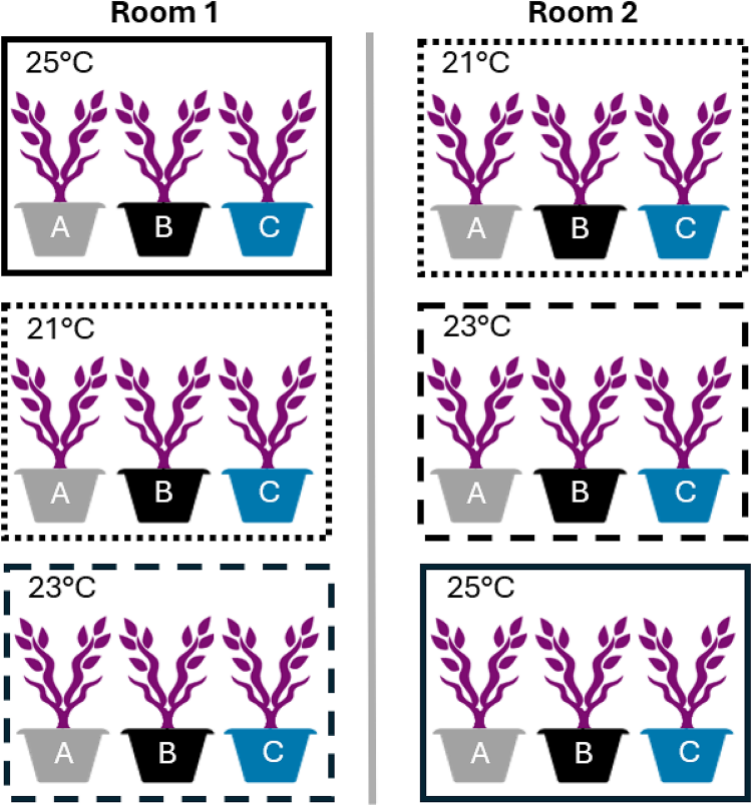
Schematic diagram of the experimental design where rectangles represent three vertically stacked growth chambers per room. Because of different physical dimensions, Room 1 housed 40 plants per chamber and Room 2 housed 30 plants per chamber. Each chamber was randomly assigned one of three temperature treatments (21, 23 or 25°C) and, within each chamber, each plant was assigned one of three fertilizer treatments (A, B or C) for a total of 70 plants per fertilizer or temperature treatment and 210 plants total.

The plants were grown in six growth chambers located across two rooms, with three stacked chambers in each room. Room one housed 40 plants per chamber and room two housed 30 plants per chamber, totaling 210 plants. Chamber dimensions were as follows: three chambers measured 254 cm x 104 cm x 102 cm, and three chambers measured 254 cm x 67.3 cm x 102 cm^33–35^ spread across two growing rooms (Figure 1). Soil moisture was maintained between 15–35%, with approximately 500 mL of water provided every two days. Water and nutrient solution pH levels were monitored and adjusted to 6.0 three times per week.^18^

### Experimental design

The experimental design included two factors: temperature and fertilizer treatments. The six chambers were assigned one of three temperature treatments (21, 23 or 25°C), with two replicate chambers per treatment. Temperature was monitored by the internal thermocouple within the growing chambers.^33–35^ The temperature treatments were applied from June 28 to November 10, 2022.

Each plant was randomly assigned a location within a chamber and one of three fertilizer treatments (A, B, or C, Appendix Table A2). Each fertilizer treatment was applied to 70 plants per temperature treatment, with a total of 210 plants across all chambers. Fertilization followed the FloraSeries^®^ 3-Part Hydroponic-Based Nutrient System (General Hydroponics Inc.), with weekly applications of 500 mL of fertilizer solution from June 29 to November 10.^18^

Fertilizer A was based on Treftz & Omaye's recipe,^18^ containing an average nutrient concentration of 500 ppm for 11 weeks. Fertilizer B used a more concentrated formula, following the weekly FloraSeries^®^ “Medium Feed” schedule.^12,26^ After 8 weeks, we repeated the week 8 nutrient recipe for the remainder of the experiment. Fertilizer C was similar to B, but included inoculation with Glomus intraradices (DYNOMYCO^®^, Volcani Centre for Agricultural Research, Rishon LeZion, Israel) mycorrhizae to test its effects on fruit production and quality. Inoculation was performed twice: the first during week 2 (0.3 g mycorrhizae + 25 g sucrose) and the second week 4 (2.1 g mycorrhizae + 25 g sucrose). Both inoculations were mixed into 500 mL of nutrient solution per plant. On Oct. 20, approximately 4 months after planting, the primocanes (fruiting canes) were pruned from all plants.

### Data collection

Stem diameter of the floricanes was measured at planting. Once the raspberries began producing ripe fruit on August 8, we harvested fruits weekly from each plant until the experiment ended on November 10th (a total of 14 weeks). Berries harvested during August, September, October and November were grouped as Month 1, 2, 3, and 4 respectively. Each harvested berry was counted, weighed using an analytical scale (Mettler-Toledo, Columbus, OH), and stored in a freezer at −20°C for later Brix analysis.

**Figure 2.**
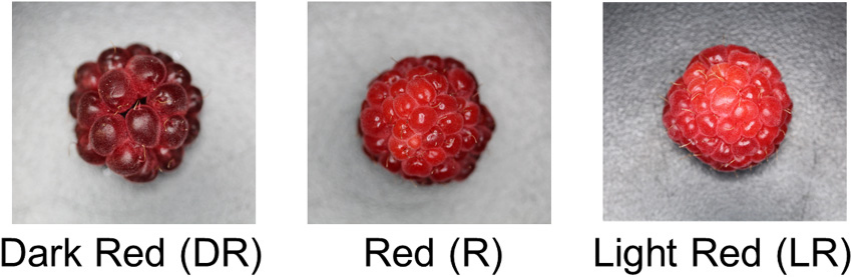
Berry colour was divided into three categories, light red (LR), red (R), and dark red (DR) and this image acted as a colour reference.

For each plant, we recorded individual berry biomass (g), total berry biomass per plant, and the number of fruits produced. Additionally, the color of each raspberry fruit was visually assessed to determine ripeness, categorized as light red, red, or dark red (Figure 2). Once harvested, raspberries were frozen in labeled, resealable plastic bags (Ziplock, San Diego, CA).To calculate the harvest index for each fertilizer treatment group, the above-ground vegetative biomass of 26 plants (only grown at 21°C) was dried at 40°C for four days, and the dry biomass of each plant was weighed. These plants included 9 from Fertilizer A, 10 from Fertilizer B, and 7 from Fertilizer C.

### Sugar content analysis

To assess the impact of temperature and fertilizer treatments on the sugar content of raspberries, we measured the Brix of bulk fruit samples from each plant, combining fruits at different ripeness stages (Figure 2). Of the 8249 fruits evaluated for their ripeness, 70% were classified as “dark red”, 25% as “red” and 5% as “light red”.

Raspberries harvested from 120 individual plants were thawed for 24 h, then bulk macerated using a pestle and metal strainer into 50 mL falcon tubes (Falcon, Somerville, MA). A calibration curve was prepared using 99% pure sucrose to ensure the accuracy of the Brix Refractometer (Cole-Parmer, Vernon Hills, IL). Then, 200 μL of raspberry juice was measured using the Brix meter. An additional 500 μL of raspberry juice was pipetted into 1.5 mL microcentrifuge tubes (Biomart Co, Brampton, Canada) of known weight and dried in an oven (LAB-LINE INSTRUMENTS, Melrose Park, Illinois) at 90–100°C for 24 h to obtain the dry matter in the juice. The total dry matter was then weighed on a microanalytical scale, and the sugar content was calculated using: sugar content = (Brix/0.651)-4.47, where the equation was derived from an experimentally determined sucrose calibration curve.

### Harvest Index calculations

To calculate the harvest index for each fertilizer treatment at 21°C, we used the total fruit biomass data. We estimated the dry biomass of fruit by multiplying the total biomass of the fruits by the dry to wet biomass ratio.^36^ The estimated dry fruit biomass was then divided by the total vegetative biomass of the plant plus the total fruit biomass to calculate the harvest index. Due to limited data, all plant data used for the harvest index calculation was from the 21°C temperature treatment.

**Table 2.** A comparison of individual fruit biomass and number of fruit harvested from ‘Joan J’ raspberry plants in each of 4 months in a vertical farm in Toronto, Canada. We performed repeated measures ANOVA for each trait over 4 months (time = within-subjects effect). Plants were grown in one of three fertilizer treatments (Appendix 1) at one of three temperatures (Temp = 21, 23, or 25°C), and in one of two rooms (block). F-statistics are presented to indicate significant differences: ns, P > 0.10; +, P < 0.10; *, P < 0.05; **, P < 0.01; ***, P < 0.001.

	Avg Fruit Biomass		Avg No. Fruit per Plant	
Source	df_N,D_	F	df_N,D_	F
Between-Subjects effects:				
Temperature (Temp)	2, 50	17.19***	2, 51	0.25
Fertilizer (F)	2, 50	1.52	2, 51	1.61
Temp x F	4, 50	1.15	4, 51	0.90
Within-Subjects Effects:				
Time	1.72, 86.1	33.33***	3153	2.19^+^
Time x Temp	3.44, 86.1	3.70*	6153	0.36
Time x F	3.44, 86.1	0.56	6153	0.49
Time x F x Temp	6.89, 86.1	0.99	12,153	0.55

### Statistical analysis

All analyses were performed using the Car package^37^ in R statistical software (v. 4.1.3, Rstudio, Bostan, MA, USA). Response variables were log transformed to adhere to assumptions of normality.

To test for differences in whole plant total fruit biomass and total number of fruits per plant, we ran a linear mixed model MANOVA where temperature and fertilizer were considered to be fixed effects, and block (the growth room) was a random effect with subsequent univariate ANOVAs when significant differences were detected. To test for differences in sugar content of berries, we ran a linear mixed model MANOVA with temperature and fertilizer as fixed effects and block as a random effect. To test for differences in number of fruit produced per month and individual fruit weight over four months, between temperature and fertilizer treatments, we ran a repeated measures ANOVA for each measured trait. Temperature and fertilizer were considered to be fixed effects, time (month) was considered a repeated measure, and block (the growth room) was a random effect. To test for differences in Harvest Index, we ran a linear mixed model ANOVA where fertilizer was the fixed effect and block (the growth room) was a random effect. Post-hoc comparisons were performed using a Bonferroni correction.

## Results

### Influence of temperature and fertilizer treatments on raspberry fruit

Of the 210 plants, 13 plants died during the experiment and 15 plants did not produce any fruit. Of the 13 plants that died, 2 were grown at 21°C, 4 were grown at 23°C and 7 were grown at 25°C; in addition, 4 were grown with A fertilizer, 7 were grown with C fertilizer and 2 were grown with B fertilizer. Of the 15 plants that did not produce fruit, 5 were grown at 21°C, 2 were grown at 23°C and 8 were grown at 25°C; moreover, 5 plants were grown with A, C and B fertilizer respectively.

**Figure 3.**
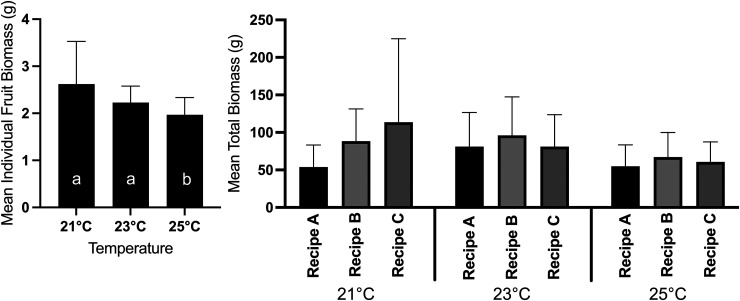
Comparison of mean (±SD) individual and total raspberry fruit biomass harvested from *Rubus idaeus*, cv. ‘Joan J’ grown in a vertical hydroponic farm at three temperatures (21, 23, or 25°C) and three fertilizer recipes (A, B or C). Statistical differences among treatments were analyzed using a linear mixed model MANOVA with temperature and fertilizer as fixed effects and block (growth room) as a random effect. Post-hoc comparisons were conducted using a Bonferroni correction (α = 0.05). N ≃ 70 per temperature treatment and N ≃ 23 per temperature by fertilizer treatment combination.

Plants produced significantly (30%) less total fruit biomass when grown at 25°C than plants grown at 21 or 23°C (Figure 3, Table 1, post-hoc mean differences > 0.13, p < 0.03) and significantly more (23%) fruit over their lifetime when grown at 23°C (Table 1, post-hoc mean differences > 9.16, p < 0.05). Moreover, plants produced significantly (26–35%) more fruit when fertilized with treatment B, relative to fertilizer A or C (Table 1, post-hoc mean differences p < 0.03).

**Table 1.** A comparison of total fruit biomass and total number of fruit harvested from ‘Joan J’ raspberry plants harvested after four months in a vertical farm in Toronto, Canada. We performed a multivariate ANOVA that included both traits (Pillai's trace method), where total fruit biomass was log_10_ transformed. Plants were grown in one of three fertilizer treatments (Appendix 1) at one of three temperatures (Temp = 21, 23, or 25°C), and in one of two rooms (block).

Source	df_H,E_	F_MANOVA_	df_H_	F_Total Fruit Biomass_	F_Total # Fruit_
Fertilizer (F)	4356	3.33*	2	2.82	5.16**
Temperature (T)	4356	6.21***	2	6.26**	3.76*
Block (B)	2177	0.51	1	0.27	0.93
F x T	8356	0.45	4	0.68	0.52
F x B	4356	1.31	2	1.46	0.15
T x B	4356	1.82	2	2.19	0.78
F x T x B	8326	1.50	4	0.46	0.46

**Figure 4.**
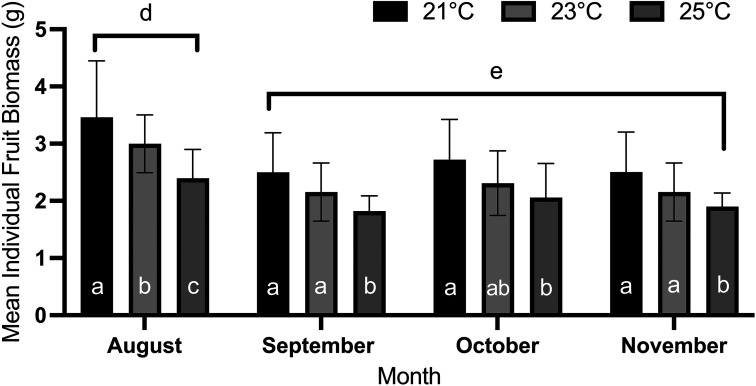
The change in the mean individual fruit biomass (±SD) for raspberry (*Rubus idaeus*, cv. ‘Joan J’) grown in a vertical hydroponic farm at three temperature treatments (21, 23, 25°C) under controlled environmental conditions between August – November 2022, as a function of time. Over a four month period, mean individual fruit biomass declined significantly through time, where berries produced in the first month possessed significantly greater average individual biomass than berries produced during any other month (repeated measures ANOVA: F_1.72, 86.1_=33.3, p < 0.001). Post-hoc comparisons were conducted using a Bonferroni correction (α = 0.05). Letters a-c connote significant differences between temperature treatments within a month. Letters d-f connote significant differences between months. Because time (month) was a within-subjects effect, temperature treatments between months cannot be compared.

**Figure 5.**
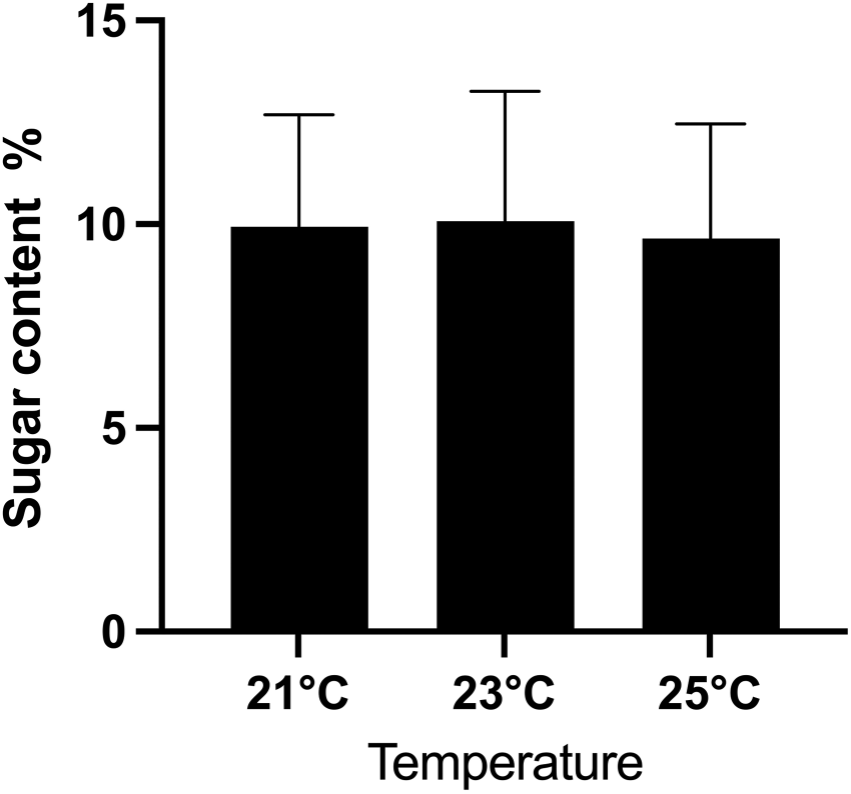
Comparison of mean sugar content (%, ± SD) of raspberry fruit harvested from *Rubus idaeus*, cv. ‘Joan J’ grown in a vertical hydroponic farm at three temperatures (21, 23, or 25°C). Statistical differences among treatments were analyzed using a linear mixed model MANOVA with temperature as a fixed effect and block (growth room) as a random effect. Post-hoc comparisons were conducted using a Bonferroni correction (α = 0.05). N ≃ 40 per temperature treatment. Letters connote significant differences between temperature treatments.

Over the four month period of the experiment, average individual fruit biomass changed significantly through time (Month 1–4; Table 2, F_1.72, 86.1 50_ = 33.33, p < 0.001), where berries produced in the first month had were significantly (on average 29%) greater average individual biomass than berries produced during any other month (Figure 4, post-hoc mean differences between Month 1 and any other month > 0.15, p < 0.001) and average individual berry biomass did not differ significantly among those harvested in Months 2–4. There was also a significant interaction between temperature and time on the effect of raspberry biomass (Table 2, F_3.44, 86.1_ = 3.70, p < 0.001) such that berries grown in Month 1 at 21°C were significantly larger than berries produced at any other time and temperature combination (Figure 5). However, there was no significant interaction between fertilizer treatment and time on the effect of raspberry biomass.

**Table 3.** A comparison of average fruit sugar content of fruit harvested from 120 ‘Joan J’ plants harvested over four months from a vertical farm in Toronto, Canada. We performed an ANOVA, where average sugar content was log_10_ transformed. Plants were grown in one of three fertilizer treatments (Appendix 1) at one of three temperatures (Temp = 21, 23, or 25°C).

Factor	Df	Sum Sq	Mean Sq	F value	P value
Fertilizer (F)	2	0.08	0.04	0.33	0.72
Temperature (T)	2	1.42	0.71	6.18	0.003**
F x T	4	0.28	0.07	0.61	0.65
Residuals	110	12.6	0.11		

### Harvest Index

**Figure 6.**
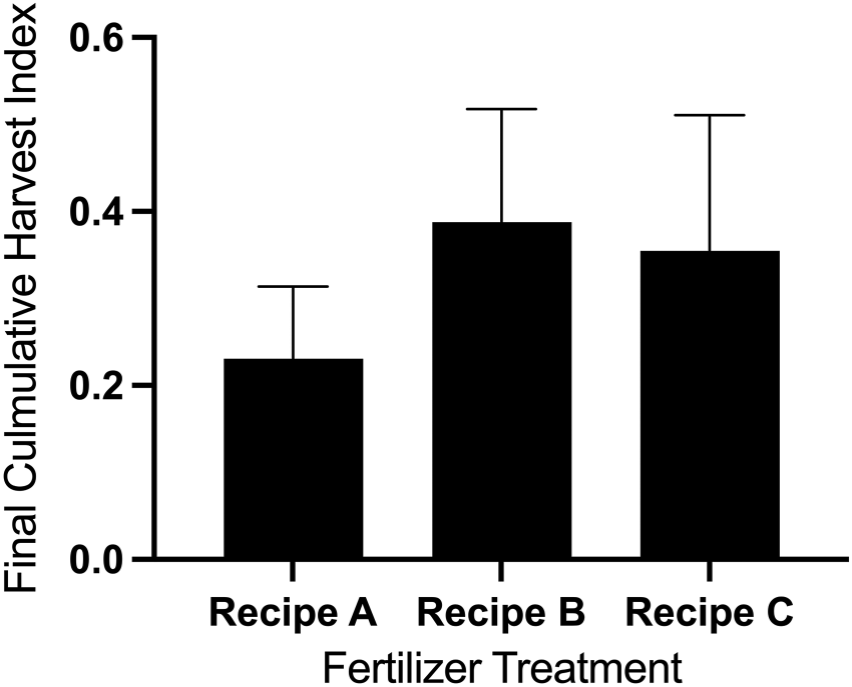
Comparison of mean harvest index (±SD) of *Rubus idaeus*, cv. ‘Joan J’ plants grown in a vertical hydroponic farm at 21°C under one of three fertilizer treatments; Recipe A: weak fertilizer applied at a constant rate, B: developmentally adjusted fertilizer (DAF) and C: DAF plus commercial endomycorrhizal fungi mixture. Statistical differences among treatments were analyzed using a linear mixed model ANOVA with fertilizer as a fixed effect and block (growth room) as a random effect. Post-hoc comparisons were conducted using a Bonferroni correction (α = 0.05). N = 7–10 plants per fertilizer treatment. Letters connote significant differences between fertilizer treatments.

On average, the harvest index of the raspberries grown at 21°C over a four month period was 33%. Plants expressed significantly different harvest indices among fertilizer treatments (F_2,24_ = 3.35, P = 0.05). Although plants with the AMF fertilizer treatment did not differ significantly with other fertilizer treatments, the plants grown with the Recipe B fertilizer treatment had significantly a higher harvest index than the plants grown under the Recipe A fertilizer treatment (Figure 6).

### Influence of temperature and fertilizer treatments on raspberry sweetness

The raspberries harvested and measured for sugar content were at different stages of ripeness, such that 70.71% were at the dark red stage, 24.42% at the red stage and 4.87% at the light red stage (Figure 2). Average sugar content across all raspberries was 9.89°Bx (9.89% sugar concentration). Average sugar content varied among the temperature treatments (Table 3, F_2_ = 6.18, P < 0.05) with plants grown at 23°C significantly sweeter fruit than plants grown at the other temperatures. There were no significant effects of fertilizer or fertilizer by temperature treatments on sugar content of fruit.

## Discussion

### Influence of temperature and fertilizer on raspberry yield

Our results corroborate previous research, demonstrating an increase in individual raspberry fruit weight with relatively low temperatures, a trend observed across diverse cultivars including ‘Polka’, ‘Erika’, and ‘Autumn Treasure’.^22,38^ The largest and sweetest fruits were produced at 23°C, which underscores the critical role of temperature regulation in hydroponic raspberry cultivation and the importance of infrastructure in optimizing fruit biomass.^14^ Across the four-month harvest period, we observed a decline in fruit weight, also consistent with previous studies, with the most significant drop occurring between Months 1 and 2, possibly due to resource limitation as plants matured.^21,22,32^ The interaction between temperature and time significantly influenced fruit weight, with larger berries initially produced at the lowest temperature, highlighting economic implications for growers seeking off-season pricing benefits.^38^ Further, raspberry plants are known to have reduced survival in hydroponic systems,^18^ and certainly, in our system, high temperatures seem to be more frequently affiliated with plant death or a lack of fruit production. Although our system couldn't reach temperatures below 21°C, further exploration of their impact on maximizing the harvest index is warranted, while our fertilizer treatments showed no effect on fruit weight, aligning with previous findings, suggesting potential benefits of multi-year studies to uncover their influence.^25^

Plants produced significantly greater total fruit biomass and higher harvest index when fertilized with relatively higher nutrient concentrations to those offered in Treftz & Omaye^18^ and plants fertilized with fertilizer B produced significantly more fruit over their lifetime than plants fertilized with the relatively weak fertilizer treatment A. Using mycorrhizae to help increase the efficient use of nutrients appears to reflect a nuanced relationship between fertilizer types and AMF's role in plant growth, potentially affecting how plants respond to different nutrient sources.^27^ Generally, AMF inoculation benefits plants by synthesizing phytohormones and secondary metabolites, solubilizing minerals, enhancing nutrient uptake, improving water absorption, increasing resilience under stress conditions, promoting osmolyte production, and enhancing soil texture.^39^ Because plants grown with fertilizer C were not stressed, our result was not particularly surprising, relative to the AMF literature. Moreover, plants grown with fertilizer C tended to die more frequently than plants grown with other fertilizers. These results indicated that specific fertilization strategies can influence overall yield efficiency in hydroponically grown raspberries. Overall, our findings contribute to understanding the complex interplay of temperature, time, and cultivation practices in optimizing raspberry fruit weight and quality.

The analysis of raspberry fruit sweetness in relation to temperature and fertilizer treatments may have been confounded by the varying ripeness stages of the sampled fruits. The presence of both overripe and under ripe raspberries in samples could have introduced significant variability in the measurements of soluble solids, leading to non-significant differences between the treatments. The ripening process significantly affects various quality attributes of raspberry fruits, including color, firmness, acidity, and sugar content.^40–43^ The accumulation of sugars and organic acids varies throughout the ripening stages, with ripe fruits typically exhibiting higher sugar concentrations compared to underripe or overripe fruits.^44^ Furthermore, the metabolic pathways responsible for flavor and sweetness are intricately linked to the fruit's maturity, suggesting that the timing of harvest relative to ripeness is crucial for accurate assessments of sweetness.^45–47^ Consequently, the mixed maturity of the fruit samples may have obscured the true effects of temperature and fertilizer treatments on sweetness, thereby increasing the error in determining soluble solids content and resulting in the observed lack of significant differences among the treatments.^48^

### Mechanisms that may explain the observed outcomes

Temperature and appropriate fertilizer regimes likely impact several mechanisms that have crucial roles in determining raspberry yield. Although higher temperatures accelerate shoot elongation and node formation during vegetative stages, and raspberry plants can technically initiate the development of flower buds at temperatures as high as 30°C, lower temperatures during flowering and fruit lead to earlier and increased flowering, which directly impacts fruit yield (Sønsteby & Heide, 2012). The optimal temperature for photosynthesis in raspberries, particularly for maximizing photosynthetic efficiency (Fv/Fm) and overall plant health, generally falls within the range of 22°C to 25°C^49^ Further, exposure to different temperatures triggers significant changes in the gene expression profiles of raspberries, sometimes attributed to the R. arcticus gene.^38^ Raspberries exposed to elevated temperatures both up-regulate and down-regulate specific genes related to stress responses, metabolic processes, and developmental pathways. These genetic responses can alter physiological traits such as growth rate, flowering time, and stress resilience, all of which contribute to yield.^49^ Interestingly, at least in the northern hemisphere, raspberries tend to produce fruit during the hottest part of the year, when temperatures are often well above 22°C to 25°C. Temperature stress likely alters various biochemical pathways and physiological responses which aim to preserve cellular function under stressful conditions but which may divert resources away from growth and fruit production, thus impacting yield and flavour.^15,20–22,38,50^

### Impact of vertical farming on raspberry production

Controlled environment agriculture is forecasted to become a prevalent method for the production of fruits and vegetables, offering year-round production opportunities in Canada and reducing reliance on imports. This is particularly relevant for raspberries, which are a significant economic contributor; in 2022, Canada exported $23 million worth of raspberries but imported $615 million worth, equating to 8.5% of all fruit imports.^3,6^ Currently, the majority of Canadian raspberries are grown outdoors or in high tunnels with limited temperature control, leading to just one extended or two short cropping cycles annually.^51^ Vertical farming, which has been increasingly adopted for leafy vegetables and herbs, presents a promising yet underutilized opportunity for raspberry production due to high domestic demand and the challenges associated with raspberry cultivation, including their sensitivity to spoilage. By providing a controlled environment, vertical farms could enable Canadians to boost local raspberry production throughout the year, satisfying domestic demand and potentially supporting exports. Unlike greenhouses or hoop-house environments that experience significant fluctuations throughout the year,^52–55^ vertical farms maintain consistent micro-climates, optimizing conditions for raspberry growth in terms of temperature and nutrient fertility.

### Sustainable raspberry production

In 2015, food systems emitted one third of all global greenhouse gas emissions^56,57^ which are largely determined by energy use, industrial activity, and waste management. Vertical farms, which currently have high energy demands, must prioritize energy efficiency and adopt decarbonization technologies in each farm. Thus, identifying environmental recipes that support consistent microclimates and extend growing seasons can increase crop yield and reduce emissions associated with transportation and land use change. This approach allows vertical farms in cities to utilize their energy demands more efficiently. Harvest indices provide us with a metric by which we can measure our efficiency with which we convert raw inputs into harvested food (e.g.,^58^ and we encourage other researchers in this field to adopt similar metrics. Vertical farming will offer the opportunity to learn new methods for growing crops like raspberries, indeed presenting new challenges. For instance, in this experiment, we learned Joan J raspberries will continue to produce fruit for at least 8 months (in contrast to outdoor grown raspberries which may fruit for 3–4 months). Determining when to replace raspberry canes (or clones of any fruiting plant) will be a new question for indoor farms. We encourage stakeholders in the vertical farm sector (including farmers, researchers and policy makers) to invest in and support the development of sustainable vertical farming initiatives for raspberries and other soft fruit. We anticipate, for instance, that fertilizer recipes could be further improved, with the careful study of the rhizosphere and the identification of endosymbiotic organisms that flourish in hydroponic culture. This type of research could increase the sustainability of vertical farming and allow local communities to become more self-sufficient in fruit production through sustainable practices.

### Conclusions & future directions

In conclusion, this study highlights the optimal growing conditions for maximizing fruit quality of red raspberries in a CEA hydroponic system. Recommendations include using a phenology-based fertilizer schedule at 21°C or 23°C to grow 'Joan J' cultivar red raspberries. Further research should focus on exploring additional factors such as spacing, irrigation, pruning techniques, and cultivar selection to establish CEA as a viable method for raspberry cultivation. Future studies could also investigate the influence of temperature and fertilizer treatments on other fruit quality parameters such as flavor and sweetness. Understanding the environmental factors that influence sugar content in raspberries could lead to the development of more efficient growing practices in hydroponic systems.

## Appendix

**Table A1.** Raspberry Light and Humidity Ramp Up Schedule.

Date	AMPL system %	PPFD (umolm-2s-1) (+/- 3)	Humidity
Jun 10, 2022	20% CW (all)		40% (day & night)
	Water: 700 ml		
Jun 13, 2022	30% CW (all)		40% (day & night)
	Water: 700 ml		
Jun 15, 2022	40% CW (all)	8	40% (day & night)
Jun 16, 2022	Water: 750 ml		
June 17, 2022	50% CW (all)	16	Day: 50% Night: 45%
	10% DR (AMPL 1)	20	
	72% DR (AMPL 2/3)		
	CW +DR	35	
Jun 20, 2022	60% CW (all)	30	Day: 55% Night 50%
	35% DR (AMPL 1)	60	
	87% DR (AMPL 2/3)		
	CW +DR	90	
	Water: 750 ml		
Jun 22, 2022	70% CW (all)	70	Day: 60% Night: 50%
	64% DR (AMPL 1)	105	
	94% DR (AMPL 2/3)		
	CW + DR	170	
	Water: 750 ml		
Jun 27, 2022	95% CW (AMPL 1)		Day: 60% Night: 60%
	90% CW (AMPL 2/3)		
	DR (AMPL 1)		
	DR (AMPL 2/3)		
	CW + DR		
	Water: 750 ml		
	Began temperature treatments		

**Table A2.** Weekly Nutrient Recipe Schedule.

**Week**	**1**	**2**	**3**	**4**	**5**	**6**	**7**	**8**	**9**	**10**	**11**
**Recipe A** Components (mg / 500 ml):											
N	17	17	17	17	17	17	17	17	17	17	17
P	9	9	9	9	9	9	9	9	9	9	9
K	51	51	51	51	51	51	51	51	51	51	51
**Recipe B** Components (mg / 500 ml):											
N	38	51	62	54	54	49	49	49	35	35	21
P	25	27	40	52	52	57	57	57	35	35	27
K	52	65	85	86	86	89	89	89	59	59	40
**Recipe C** Components (mg / 500 ml):											
N	38	51 + M (0.3g/plant)	62	54 + M (2.1g/plant)	54	49	49	49	35	35	21
P	25	27	40	52	52	57	57	57	35	35	27
K	52	65	85	86	86	89	89	89	59	59	40

A: Recipe A over 11 weeks based on work completed by Treftz & Omaye (2015); B: Recipe B over 11 weeks. Each plant received approximately 500 ml of this solution 3 days per week. Note that once we reached week 11, we continued with that recipe indefinitely; C: Recipe B with added mycorrhizae (+M in weeks 2 and 4) plus sucrose added twice in solution over 11 weeks. Each plant received approximately 500 ml of this solution 3 days per week. Once we reached week 11, we continued with that recipe until the end of the experiment.
